# Effect of Elatol, Isolated from Red Seaweed *Laurencia dendroidea,* on *Leishmania amazonensis*

**DOI:** 10.3390/md8112733

**Published:** 2010-10-29

**Authors:** Adriana Oliveira dos Santos, Phercyles Veiga-Santos, Tânia Ueda-Nakamura, Benedito Prado Dias Filho, Daniela Bueno Sudatti, Éverson Miguel Bianco, Renato Crespo Pereira, Celso Vataru Nakamura

**Affiliations:** 1 Programa de Pós-graduação em Microbiologia, Universidade Estadual de Londrina, Rodovia Celso Garcia Cid, PR 445, Km 380, CEP 86051-990, Campus Universitário, Londrina, Paraná, Brazil; E-Mails: oi_dri@hotmail.com (A.O.S.); bdpfilho@uem.br (B.P.D.F.); 2 Programa de Pós-graduação em Ciências Farmacêuticas, Laboratório de Inovação Tecnológica no Desenvolvimento de Fármacos e Cosméticos, Bloco B-08, Universidade Estadual de Maringá, Av. Colombo 5790, CEP 87020-900, Maringá, Paraná, Brazil; E-Mails: phercyles@gmail.com (P.V.-S.); tunakamura@uem.br (T.U.-N.); 3 Departamento de Biologia Marinha, Universidade Federal Fluminense, Caixa Postal 100644, CEP 24001-970, Niterói, Rio de Janeiro, Brazil; E-Mails: dbsudatti@gmail.com (D.B.S.); egbrecp@vm.uff.br (R.C.P.); 4 Programa de Pós-graduação em Química Orgânica, Universidade Federal Fluminense, Outeiro de São João Baptista, s/n, CEP 24.020-150, Niterói, Rio de Janeiro, Brazil; E-Mail: ebianco@chemist.com (É.M.B.)

**Keywords:** antileishmanial activity, *Leishmania amazonensis*, *Laurencia dendroidea*, elatol

## Abstract

In the present study, we investigated the antileishmanial activity of sesquiterpene elatol, the major constituent of the Brazilian red seaweed *Laurencia dendroidea* (Hudson) J.V. Lamouroux, against *L. amazonensis.* Elatol after 72 h of treatment, showed an IC_50_ of 4.0 μM and 0.45 μM for promastigote and intracellular amastigote forms of *L. amazonensis*, respectively. By scanning and transmission electron microscopy, parasites treated with elatol revealed notable changes compared with control cells, including: pronounced swelling of the mitochondrion; appearance of concentric membrane structures inside the organelle; destabilization of the plasma membrane; and formation of membrane structures, apparently an extension of the endoplasmic reticulum, which is suggestive of an autophagic process. A cytotoxicity assay showed that the action of the isolated compound is more specific for protozoa, and it is not toxic to macrophages. Our studies indicated that elatol is a potent antiproliferative agent against promastigote and intracellular amastigote forms, and may have important advantages for the development of new anti-leishamanial chemotherapies.

## 1. Introduction

*Leishmania amazonensis*, a flagellated protozoan parasite, is the causative agent of human cutaneous leishmaniasis. In this infectious disease, a high proportion of cases evolve to severe anergic diffuse cutaneous leishmaniasis, which is severely debilitating and disfiguring [1,2]. Because of the devastating consequences to the patient, it is recognized as a special public health problem [3]. Cutaneous leishmaniasis is endemic in 88 countries on five continents, with 1–1.5 million cases reported yearly worldwide [4,5]. Poor nutrition, infection, and other stresses predispose patients to increased morbidity and mortality. Moreover, cases of *Leishmania* and human immunodeficiency virus coinfection have recently increased [6,7]. No vaccines for preventing infection are currently available [8,9].

Pentavalent antimonial compounds, sodium stiboglucanate (Pentostam), and meglumine antimoniate (Glucantime), have been recommended as the first line of drugs in the treatment of cutaneous leishmaniasis for 50 years. However, these substances cause toxic effects, including nausea, vomiting, diarrhea, skin eruptions, headache, dizziness, cardiac arrhythmia, and hypotension [10–12]. In cases of resistance, amphotericin B and Pentamidine are the main alternative drugs, but their frequent and severe side effects limit their use. There is an urgent need for new and more efficient therapies for leishmaniasis.

Elatol was isolated from *Laurencia elata* for the first time by Sims *et al.* [13]. Various species of *Laurencia* (order Ceramiales, family *Rhodomeleceae*) also produce the sesquiterpene elatol as its major secondary metabolite [14–19]. Several studies have shown that elatol plays important roles in ecological interactions, such as antiherbivore activity and potential defense against infection by microorganisms [13–16]. Recent studies have shown that elatol can be synthesized in the laboratory [20,21]. Therefore, this study investigated the antileishmanial activity of elatol isolated from the Brazilian red seaweed *Laurencia dendroidea* against the promastigote and intracellular amastigote forms of *L. amazonensis.* Additionally, we used electron microscopy techniques to evaluate the effect of elatol on the morphology and ultrastructure of the parasite.

## 2. Results and Discussion

**Figure 1:**
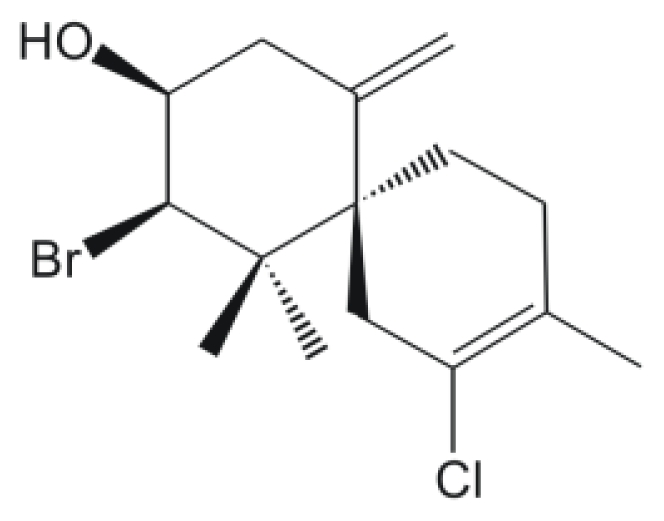
The chemical structure of elatol.

Marine algae have been used in traditional remedies in Asian countries including China, Japan, and Korea [22]. Species of *Laurencia* (order Ceremiales, family Rhodomeleceae) have proved to be a rich source of halogenated secondary metabolites, predominantly sesquiterpenes, diterpenes, and C_15_ non terpenoids [23–25]. Probably, these halogenated metabolites defend against marine herbivores and infection by microorganisms [23,26–28]. Various studies have reported that seaweeds have shown important biological activities, including antibacterial, antifungal, antileishmanial, antitrichomonal, antihelmintic, antiviral, antipyretic, analgesic, anti-inflammatory, antioxidative, and anticoagulant [15,20,22,25,28–36]. In the present study, we assessed the antileishmanial activity of sesquiterpene elatol, the major constituent of the Brazilian red seaweed *L. dendroidea* (Hudson) J.V. Lamouroux, against *L. amazonensis.* The physical and spectroscopic properties of the sesquiterpene elatol (Figure 1) were identical with data previously reported by König and Wright [14].

**Figure 2:**
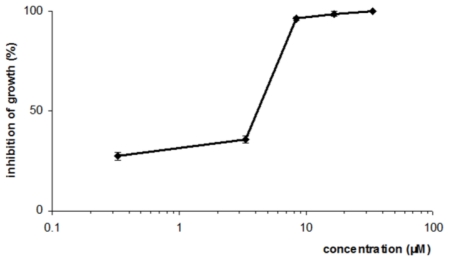
Effect of elatol against promastigote forms of *L. amazonensis*.

Elatol showed a dose-dependent antileishmanial activity against the promastigote forms. Concentrations of 4.0 ± 0.3 μM and 7.5 ± 0.5 μM of elatol induced 50% and 90% growth inhibition on *L. amazonensis*, respectively, after 72 h of treatment (Figure 2).

**Figure 3:**
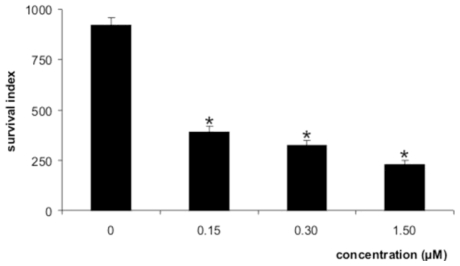
Survival index of *L. amazonensis* within peritoneal macrophage cells treated with elatol. * Significant difference of each group from the control (p < 0.05).

Figure 3 shows the effects of the elatol on the *L. amazonensis*-macrophage interaction. When the parasites were treated with elatol, a dose-dependent decrease of intracellular amastigotes was observed. The survival index was calculated as 392.0 for 0.15 μM; 325.0 for 0.30 μM; and 230.0 for 1.5 μM. These results correspond to rates of inhibition of survival as high as 57, 65, and 75%, respectively. The IC_50_ for the intracellular amastigote form was 0.45 μM. The intracellular forms are a great challenge in the treatment of leishmaniasis. All the results were significant at *p* ≤ 0.05 compared to the control group, by Student’s *t*-test. Amphotericin B showed IC_50_ of 0.06 μM and 0.31 μM against the promastigote and intracellular amastigote forms, respectively. Elatol was also evaluated for its potential toxic effects on macrophage strain J774G8. When macrophages were treated with elatol, the 50% cytotoxic concentration (CC_50_) was 1.4 μM. The toxicity to the macrophage and the activity against protozoa were compared by using the selectivity index (SI) (ratio: CC_50_ macrophage J774G8 cells/IC_50_ protozoa). The observed SI for the intracellular amastigote forms was 3.0 times less toxic to the macrophage than to the protozoa.

**Figure 4:**
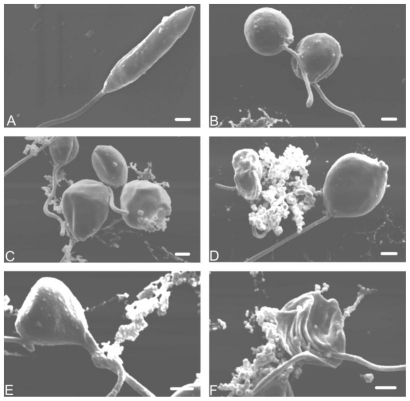
Scanning electron microscopy of promastigote forms of *L. amazonensis* treated with elatol after incubation for 48 h at 25 °C. (**A**) Control; (**B**–**F**) Parasites after treatment with IC50 of elatol. Bars = 1 μm.

By scanning electron microscopy, promastigote forms treated with elatol revealed notable morphological changes compared with the control cells (Figure 4A). There were changes in size and shape (Figure 4B, C, D, and F). In addition, cells showed rupture of the plasma membrane with loss of their contents (Figure 4C–F) and cell shrinkage (Figure 4F).

**Figure 5:**
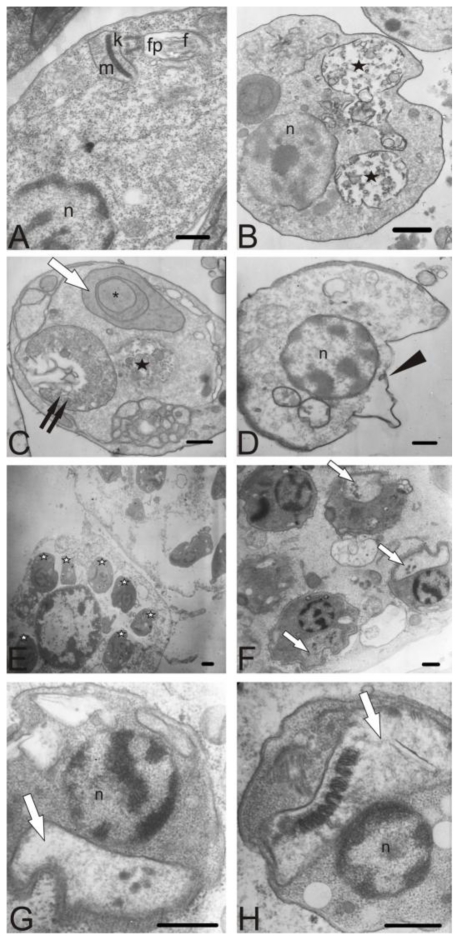
Ultrastructural effect of elatol after incubation for 48 h at 25 °C on promastigote and intracellular amastigote forms of *L. amazonensis*, observed by transmission electron microscopy. (**A**) Promastigote control; (**B**–**D**) Promastigote treated with IC50 of elatol; (**E**–**H**) Intracellular amastigote forms treated with IC50 of elatol. Elatol treatment led to swelling of the mitochondria (white arrow), autophagic vacuoles (black stars), appearance of concentric membrane structures inside the organelle (asterisk), destabilization of the plasma membrane (arrowhead), extension of the endoplasmic reticulum (two arrows), and intracellular amastigotes in peritoneal macrophages (white star). n: nucleus; f: flagellum; fp: flagellar pocket; k: kinetoplast; m: mitochondrion; Bars = 1 μm.

Photomicrographs from transmission electron microscopy are shown in Figure 5. Control parasites showed no changes in the plasma membrane, and an apparently normal ultrastructure (Figure 5A). In promastigote forms, the treatment with elatol led to a pronounced swelling of the mitochondrion and the appearance of concentric membrane structures inside the organelle (Figure 5C), destabilization of the plasma membrane (Figure 5D), and autophagic vacuoles (Figure 5B). The treated cells also formed membrane structures, apparently an extension of the endoplasmic reticulum, which is suggestive of an autophagic process (Figure 5C). A similar structure was not seen in untreated cells. We also observed the effects of elatol on intracellular amastigote forms. Peritoneal macrophages were infected in multiples of 10 promastigotes per host cell, and were incubated at 37 °C in 5% CO_2_ atmosphere. After 24 h, the infected macrophages were treated with elatol. Figure 5E shows several intracellular amastigotes in peritoneal macrophages. In the ultrastructural evaluation of treated amastigotes, the most prominent effect was swollen mitochondria (Figure 5E, F and G).

Interestingly, a similar mechanism is seen during treatment with ergosterol biosynthesis inhibitors. Lazardi *et al.* [37] showed that epimastigote and amastigote forms of *Trypanosoma cruzi* treated with the minimum growth-inhibitory concentration of ICI 195,739 (0.1 μM) displayed almost immediate ultrastructural alterations, consisting of membrane lesion, intense swelling of the mitochondrion with loss of the inner membrane, and changes in the matrix electron density, as well as the appearance of autophagic vacuoles. Similar results have been reported by Rodrigues *et al.* [38] in *L. amazonensis* promastigotes treated with azasterols, known inhibitors of the Δ^24(25)^-sterol methyltransferase, showing several alterations in the mitochondrion structure such as disorganization of the internal membranes and an intense and evident mitochondrial swelling with loss of the matrix content. This is in agreement with Lorente *et al.* [39], who found similar alterations when parasites were treated with azasterols. These compounds were shown to have ultrastructural effects on *L. amazonensis* promastigote membranes, including the plasma and mitochondrial membranes, and the endoplasmic reticulum. In addition, Rodrigues *et al.* [40] described the effects of sterol methenyl transferase inhibitors (SMTI), essential enzymes for sterol biosynthesis, on promastigote and axenic amastigote forms of *L. amazonensis.* Ultrastructural alterations in treated cells were observed mainly in the mitochondrion, which displayed intense swelling and reduced electron density of the matrix, with marked changes in the inner mitochondrial membranes.

## 3. Experimental Section

### 3.1. Plant material and extraction procedures

Specimens of *L. dendroidea* were collected by hand during low tide, in the midlittoral zone on the rocky coast of Cabo Frio Island (22°59′ S, 42°59′ W), Rio de Janeiro State, Brazil. This seaweed was previously described as *L. obtusa*, but through molecular techniques, it was recently identified as *L. dendroidea* [41]. The seaweed was stored in plastic bags and chilled on ice during transport to the laboratory. The specimens of *L. dendroidea* used in this study were identified by Dr. Mutue Toyota Fujii, and voucher specimens were deposited in the herbaria SP, Instituto de Botânica, São Paulo State, Brazil (SP number: 399789). *L. dendroidea* was dried in the dark at room temperature.

### 3.2. Extraction procedures and elatol isolation

The air-dried algal material (300.0 g) giving 50 mg of elatol was successive and exhaustively extracted in *n*-hexane at room temperature for 15 days. The *n*-hexane crude extract (HE) was evaporated to dryness on a rotary evaporator at low temperature (<50 °C), yielding 3.64 g of a dark green extract containing the sesquiterpene elatol, which was detected as brown spot on TLC plates after spraying with a solution of ceric sulphate and sulfuric acid (2.1 g of Ce_2_(SO_4_)_3_ · 4H_2_O; 21 mL of H_2_SO_4_ and 300 mL of H_2_O), followed by heating at 100 °C for 3 min. An aliquot of HE (0.35 g) was submitted to preparative thin layer chromatography (PTLC) (Merck, silica gel 60 F_254_, 20 × 20 cm, mobile phase: *n*-hexane/ethyl acetate 8:2), to afford a yellowish oil (50 mg) which was identified as the sesquitepene elatol. The purity was confirmed by TLC (Rf = 0.45), using *n*-hexane/AcOEt 8:2 as mobile phase, and by ^1^H-NMR spectroscopy (300 MHz).

### 3.3. Spectroscopic data

The physical and spectroscopic properties of the isolated elatol were identical to those previously reported by König and Wright [42].

### 3.4. Parasite and cell culture

Promastigote forms of *L. amazonensis* (MHOM/BR/75/Josefa strain) were maintained by weekly transfers in Warren’s medium [43] supplemented with 10% heat-inactivated fetal bovine serum (FBS) at 25 °C in a tissue flask. The macrophage lineage (J774G8) was maintained in tissue flasks in RPMI 1640 medium (Gibco Invitrogen Co., Grand Island, New York, U.S.) with L-glutamine and supplemented with 10% inactivated fetal bovine serum at 37 °C in a 5% CO_2_–air mixture.

### 3.5. Antileishmanial activity

*L. amazonensis* promastigotes (1 × 10^6^ parasites/mL) were inoculated in a 24-well plate containing Warren’s medium supplemented with 10% inactivated fetal bovine serum with different concentrations of elatol (0.1 to 100 μM), and incubated at 25 °C for 72 h. The cell density for each concentration was determined by counting in a hemocytometer (Improved Double Neubauer). Amphotericin B was used as a positive control. Controls containing 1.0% dimethyl sulfoxide (DMSO; Sigma Chemical Co., St. Louis, Missouri, U.S.) and medium alone were also included. The results were expressed by IC_50_ (concentration that inhibited 50% parasite growth).

### 3.6. Activity against intracellular amastigotes

After 72 h of inoculation of 5% thioglycolate medium, resident peritoneal cells from the BALB/c mice were harvested in RPMI 1640 medium (Gibco Invitrogen Corporation, New York, U.S.) pH 7.6. Cells were plated on coverslips (diameter 13 mm) in 24-well plates and allowed to adhere for 24 h at 37 °C in 5% CO_2_ atmosphere. Macrophages were infected in multiples of 10 promastigotes per host cell and were incubated at 37 °C in 5% CO_2_ atmosphere. After 24 h, infected macrophages were treated with different concentrations of elatol (0.15 to 1.50 μM). Next, the monolayers were washed with PBS at 37 °C, fixed in methanol, and stained with Giemsa. The number of amastigotes was determined by counting at least 200 macrophages in duplicate cultures, and the results were expressed as the survival index. The survival index was obtained by multiplying the percentage of infected macrophages by the number of amastigotes per infected macrophage.

### 3.7. Cytotoxicity assay

Adherent J774G8 macrophage cells in the logarithmic growth phase were suspended to yield 10_5_ cells/mL in RPMI 1640 medium supplemented with 10% FBS, and added to each well in 96-well microtiter plates. The plates were incubated in a 5% CO_2_–air mixture at 37 °C to obtain confluent cell growth. After 24 h, the medium was removed and the cells were treated with elatol (0.1 to 100 μM). Control wells without elatol were included. The plates were incubated in a 5% CO_2_–air mixture at 37 °C for 48 h. The cultures were then fixed with 10% trichloroacetic acid for 1 h at 4 °C, stained for 30 min with 0.4% sulforhodamine B (SRB) in 1% acetic acid and subsequently washed five times with deionized water. Bound SRB was solubilized with 200 μL 10 mM unbuffered Tris-base solution. Absorbance was read in a 96-well plate reader (BIO-TEK Power Wave XS) at 530 nm. Dose-response curves were plotted (values expressed as percentage of control optical density) and CC_50_ values (50% cytotoxicity concentration) were estimated by regression analysis.

### 3.8. Electron microscopy

Promastigote and intracellular amastigote forms were treated with IC_50_ of elatol at 25 °C for 24 and 48 h, respectively. The parasites were fixed in 2.5% glutaraldehyde in 0.1 M cacodylate buffer, pH 7.2. For transmission electron microscopy, cells were postfixed for 40 min in a solution containing 1% OsO_4_ and 0.8% potassium ferrocyanide in 0.1 M cacodylate buffer, washed in the same buffer, dehydrated in acetone, and embedded in Epon. Ultrathin sections were stained with uranyl acetate and lead citrate, and were observed in a Zeiss 900 electron microscope. For observation by scanning electron microscope, promastigotes were placed on a specimen support with poly-L-lysine, dehydrated in graded ethanol, critical-point dried in CO_2_, coated with gold, and observed in a Shimadzu SS 550 SEM.

### 3.9. Statistical analysis

The means and standard deviations were determined from at least three experiments. All tests were done in duplicate. Statistical analysis was performed with the program GraphPad Prism 4 (GraphPad Software, San Diego, California, U.S.). Student’s *t* test was applied, and a *p* value less than 0.05 was regarded as significant.

## 4. Conclusions

Our studies indicated that elatol is a potent antiproliferative agent against promastigote and intracellular amastigote forms of *L. amazonensis*, and induced notable changes in the ultrastructure of the mitochondrion of the parasite. Laboratory synthesis and the possibility of modifying the chemical structure of elatol may lead to important advances in the development of new anti-leishmanial chemotherapies. More *in vitro* and *in vivo* studies will be carried out to increase understanding of the mode of action of this drug and its future utilization in the treatment of leishmaniasis.
